# Study of tumor growth indicates the existence of an “immunological threshold” separating states of pro- and antitumoral peritumoral inflammation

**DOI:** 10.1371/journal.pone.0202823

**Published:** 2018-11-02

**Authors:** Antonio Brú, David Gómez-Castro, Luis Vila, Isabel Brú, Juan Carlos Souto

**Affiliations:** 1 Applied Mathematics Department, Universidad Complutense de Madrid, Madrid, Spain; 2 Instituto de Matemática Interdisciplinar, Universidad Complutense de Madrid, Madrid, Spain; 3 Laboratory of Angiology, Vascular Biology and Inflammation, IIB-Sant Pau, Hospital de la Santa Creu i Sant Pau, Barcelona, Spain; 4 Centro de Salud La Estación, Talavera de la Reina, Spain; 5 Department of Hematology, IIB-Sant Pau, Hospital de la Santa Creu i Sant Pau, Barcelona, Spain; 6 Institut Josep Carreras contra la Leucemia, Barcelona, Spain; Northern University, UNITED STATES

## Abstract

**Background:**

Peritumoral inflammation—a response mainly involving polimorphonuclear neutrophils—has traditionally been thought protumoral in its effects. In recent years, however, a number of studies have indicated that it may play an important antitumoral role. This discrepancy has been difficult to explain.

**Methods and findings:**

This work describes a tool for simulating tumor growth that obeys the universal model of tumor growth dynamics, and shows through its use that low intensity peritumoral inflammation exerts a protumoral effect, while high intensity inflammation exerts a potent antitumoral effect. Indeed, the simulation results obtained indicate that a sufficiently strong antitumoral effect can reverse tumor growth, as has been suggested several times in the clinical literature.

**Conclusions:**

The present result indicate that an ‘immunological threshold’ must exist, marking the boundary between states in which peritumoral inflammation is either harmful or beneficial. These findings lend support to the idea that stimulating intense peritumoral inflammation could be used as a treatment against solid tumors.

## Introduction

Mortality rates associated with cancer remain very high; by the end of the decade the disease will still likely cause 10 million deaths per year [1]. Current research into immunological treatments for cancer focuses on harnessing the adaptive immune response and the power of T lymphocytes. The innate immune response appears to have been neglected, yet it provides the body’s first line of defense against infections. With respect to tumors, however, the effects of polimorphonuclear neutrophils (PMNs) appear contradictory. For example, in chronic peritumoral inflammation they appear to have protumoral effects [2], but if this inflammation is acute (either natural or induced) their behavior appears to be antitumoral [3]. This has been difficult to explain, and despite recent literature reviews insisting on the antitumoral potential of these cells [3–5], peritumoral inflammation is still largely regarded as favoring tumor growth in the clinical setting. The present paper shows how the contrasting behavior of PMNs can be explained in terms of the recently established universal model of tumor growth dynamics (UMTGD) [6, 7]. This states that tumor growth satisfies molecular beam epitaxy (MBE) class dynamics [7]. Thus, 1) the growth of the tumor radius is linear in time (except at the very beginning, during which time it is exponential), 2) proliferating cells are only found at the tumor border, and 3) tumor growth is governed by the availability of space (produced by the lytic action of tumor cells against the host tissue) at the tumor border into which new tumor cells can migrate and settle [6, 7]. This model also suggests that if the space they seek is made unavailable, tumor growth must stop. PMNs, which are naturally drawn to tumors and congregate around them [7], are candidate competitors for this space—a candidacy made stronger by the fact that they are unaffected by the lytic enzymes and other degrading products produced by tumor cells [8]. When PMNs arrive in sufficient quantity (acute inflammation) they should successfully occupy this space and ‘package’ the tumor. The pressure of many layers of PMNs on the cells first arriving can then keep all possible space filled, preventing any further tumor growth. If they arrive in small numbers, however, no such packaging may occur. Indeed, this may have a protumoral effect owing to their inherent tissue-degrading action opening up further space into which the tumor can grow. This argument forms the basis of the hypothesis of the present work, which, through the use of a new UMTGD-compliant simulation tool, provides evidence of an immunological threshold, above which peritumoral inflammation is sufficient to cause antitumoral effects, but below which protumoral effects are induced.

## Materials and methods

The approval of the ethical committee of the Hospital de la Santa Creu i Sant Pau was not necessary for this project since no intervention was performed on any human subject, no biological sample was stored and the anonymity of all donors was maintained. However, all voluntary donors signed their informed consent. All samples were accessed anonymously by authors. Any patient information was known by authors. The treating physyician was MD Juan Carlos Souto at Sant Pau Hospital, Barcelona, Spain.

### Construction of the simulation tool

A simplified off-lattice tool, written in C++ and using the open source bullet physics library [9], was developed to simulate tumor growth.

**Fig 1:**
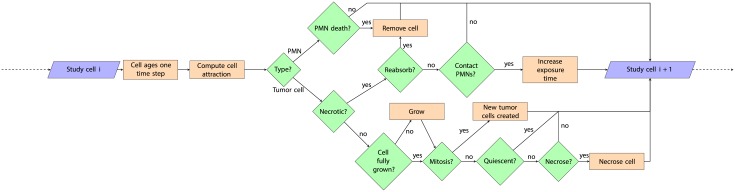
Flow diagram showing how the simulation tool works. PMN: polimorphonuclear neutrophil.

Fig 1 shows how the tool determines the fate of tumor cells under different growth conditions, starting from the founding cell in a tumor. Designed to be UMTDG-compliant, it contemplates that all tumor cells must be in one of the following states: 1) Proliferative. A state in which tumor cells can duplicate because space is available into which new cells can diffuse and settle. The time that elapses before cells undergo mitosis is normally distributed, which can be described by the following expression N(*μ*_*mit*_, *σ*_*mit*_). The tool assumes new cells to have half the radius of the parent cell, and to grow continuously over time at a constant rate until duplication size is reached once more. 2) Quiescent. A state in which tumor cells cannot duplicate because of a lack of space into which to diffuse and settle—but in which duplication may occur if the necessary space becomes available. 3) Necrotic. A state in which, after a given time in the quiescent state, cells undergo necrosis. The tool allows the visualization of the proliferative, quiescent and necrotic cells in green, red and black respectively. The tool also assumes cell-cell chemoattraction to lie behind both the formation of a tumoral mass (with tumor cells preferring to adhere to one another rather than to other host cells [10]) and the arrival around it of activated PMNs, attracted by the tumor’s production of proinflammatory cytokines [11]). This chemoattraction can be described by the expression,

$$F_{i,j}=\frac{C_{F}}{r^{2}},$$

where *r* is the distance between cells, and *C*_*F*_ is a constant. This type of equation is routinely used to describe gravitational and electromagnetic attraction. Adhering to the idea that tumor growth dynamics are universal and belong to the MBE class, the tool contemplates the existence of a strong correlation between microenvironmental space, pressure and cell proliferation. For tumor growth to be stopped, all actively dividing cells (which occur only at the tumor border) must be prevented from diffusing away from their starting positions. Thus, all the space available to them must be occupied—but this occupation must be undertaken by cells that are not affected by the lactic acid or metalloproteinases produced by tumor cells, i.e., the elements by which tumors attack host tissue and make space available [12]. Interestingly, PMNs are able to resist the actions of these agents [13]. Thus, it is contemplated that a tumor cell’s duplication is inhibited if there are more than *N*_inh_ cells (either PMNs or indeed neighboring tumor cells) at a distance of less than a specified value *R*_inh_, and that an inhibited tumor cell becomes necrotic if it remains undivided for longer than a given period *μ*_*nec*_.

#### Arrival of PMNs at the tumor border

The recruitment of PMNs to a tumor involves a series of coordinated interactions between the endothelial cells of the blood vessels in the vicinity of the tumor and the PMNs themselves [14]. The endothelial cells are activated by proinflammatory agents secreted by the tumor [15], and begin to express adhesion molecules and catechins, causing PMNs to accumulate, begin the process of diapedesis, and finally move towards the tumor. The simulation tool assumes that neutrophils arrive from a position of effective infinity, from a random direction, and at varying but constant rates under different conditions, and that this response begins at the time point “*start*_*IR*”. If a tumor was lodged in a host’s organ, migration of PMNs to the tumor would occur by extravasation. Normal vasculature away from the tumor can be considered uniformly distributed, and so arrival of PMNs from random directions is a reasonable assumption. When no external agent is detected, PMN density in the blood flow can be considered constant. Thus, the rate of extravasation can also be considered constant, and this can be translated to the model as a constant rate of arrival of PMNs. The innate immune response to external agents (bacteria, foreign bodies…) consists of an increase of PMN density. Since this incremental behaviour is not widely understood, we model this behaviour as a higher constant rate of arrival.

Activated PMNs survive for up to 12 h [16] and the tool contemplates a normal lifetime distribution for them as described by the expression *N*(*μ*_life PMN_, *σ*_life PMN_), where *μ* represents the average lifetime of a PMN and *σ* its standard deviation. Dead neutrophils are deemed to be reabsorbed by the host (at a constant rate) and therefore to disappear.

#### Phagocytosis

Once the contact time between PMNs and tumor cells unable to move into new space is greater than a fixed limit *t*_phag_, those tumor cells are assumed to become necrotic and phagocytosed by the host at a fixed rate.

#### Confirmation that the simulation tool is UMTGD-compliant: Scaling analysis of the borders of simulated tumors

Confirmation that the simulation tool is UMTGD-compliant is provided by analyzing the spatial and temporal invariances of the tumor border—exactly the same as would be done to confirm the same in real tumors or in vitro tumor cell colonies. This was performed following the methods of Bru et al. [7]. In the necessary calculations, the width of the tumor border is defined as the sum of the fluctuations around the mean position of the border. Now, by way of example, and understanding N be an integer, let us consider the points on the tumor border *x*_*i*_, where *i* = 0 to *N*. The radii of the tumor at these points take the form (*x*_*i*_, *r*_*i*_). Now, let (*r*_*i*_(*t*))_*i*=1,⋯,*N*_ be a sequence of real numbers representing these radii, that changes over time. The roughness of the border at a given time t, is thus the mean variation around the mean value for the tumor radius. This roughness, denoted by *w*(*l*, *t*), can then be expressed as:

$$\begin{array}{c} w\left(l,t\right)={\left(\frac{1}{M}\sum_{i=1}^{M}V_{i}\left(t\right)\right)}^{\frac{1}{2}}, \end{array}$$

$$\begin{array}{c} V_{i}\left(t\right)=\frac{1}{\frac{l}{N}}\sum_{j=1}^{l}{\left(r_{j+\frac{i}{N}l}\left(t\right)-m_{i}\left(t\right)\right)}^{2}, \end{array}$$

$$\begin{array}{c} m_{i}\left(t\right)=\frac{1}{\frac{l}{N}}\sum_{j=1}^{l}r_{j+\frac{i}{N}l}\left(t\right), \end{array}$$

where *m*_*i*_ is the mean value of the tumor radius in each time interval of length *l*, and *V*_*i*_ is the variation of that interval. Now, given the fractal nature of tumor borders (see [6]), their roughness varies both spatially and temporally following the power law:

$$\begin{array}{c} w\left(l,t\right)∼\{\begin{array}{cc} t^{\beta } & \text{if}\;t\ll t_{s},\\ l^{\alpha_{loc}} & \text{if}\;t\gg t_{s}, \end{array} \end{array}$$

where *α*_*loc*_ is the critical exponent of the local width, and *β* is the critical dynamic exponent of growth. Although this description is valid for many systems, in some cases the local width w(l, t) differs from the global width w(L, t) and a new critical exponent, *α*_*glob*_, must therefore be defined, which has the properties:

$$\begin{array}{c} w\left(l,t\right)∼l^{\alpha_{loc}},\;w\left(L,t\right)∼L^{\alpha_{glob}},\;t\gg t_{s}. \end{array}$$

To determine *α*_*glob*_, the power spectrum *S* (i.e., the square of the modulus of the Fourier transformation of the radii of the tumor border described by the function *r*(*x*, *t*), where $S\left(q,t\right)=|\hat{r}{\left(q,t\right)|}^{2}$ and q represents the momenta) must first be calculated. *α*_*glob*_ can then be determined from the equation below.

$$\begin{array}{c} S\left(q,t\right)∼\{\begin{array}{cc} q^{-(2\alpha_{glob}+1)}, & q\gg 1,\\ const, & q\ll 1. \end{array} \end{array}$$

Once *α*_*glob*_, *β* and *α*_*loc*_ are known, the dynamics of the simulated tumor can be determined using the method described in [6].

### Simulations

Different types of simulation were performed as described below. Table 1 shows the general values assumed for different variables.

**Table 1:** List of parameters used in the simulations.

Parameter	Model Values
*μ*_mit_	25 h
*σ*_mit_	2.5 h
*C*_*F*_	1
*N*_act_	38
*R*_act_	50.0
*μ*_*nec*_	200.0 h
*μ*_life PMN_	5 h
*σ*_life PMN_	0.5 h
arrival of PMNs	0–66 PMN/h
*start*_*IR*	200h
*t*_phag_	75 h
*max*_*radius*	1.0
*growth*_*rate*	10 radius / h
*v*_*apop*_	0.01 radius / h

The mitosis (*μ*_mit_, *σ*_mit_) are well established. The force (*C*_*F*_) and inhibition (*N*_act_,*R*_act_) parameters are fitted so that the dinamical behaviour is congruent with experiments. In particular we check that the simulation reproduces the scaling critical exponents in [7] and that the sizes of the active, inhibited and necrotic layer fit the experiments (compare Fig 3 with [7]).

#### Free growth

This simulations was performed in the absence of all interactions with PMNs in order to confirm that the tumor growth simulated obeyed the UMTGD [7].

#### Simulation of tumor-PMN interactions at different intensities of immune response

To determine how different intensities of interaction between a tumor and PMNs would affect tumor growth, the intensity of PMN arrival was varied. Tumors were simulated under free growth conditions for 214 h (i.e., to a tumor size of slightly under 200 cells) before simulated immune responses were launched at PMN arrival rates of 7-66/h for 300 h.

#### Treatment simulation

In this simulation, a tumor of the size mentioned above was subjected to a PMN arrival rate of 13 cells/h (a deficient immune response according to the results of the above simulations) for 300 h. A simulated intent to treat was then performed, changing the PMN arrival rate to 66 cells/h for the next 600 h.

The tool provided visualizations of the proliferative (green), quiescent (red) and necrotic (black) cells in all simulations. Videos were also recorded for all simulations run.

### Biological comparisons

Tumor cell colonies, which have the same growth dynamics as solid tumors [7], were prepared in order to provide biological contrast for the simulated arrival of PMNs at the tumor border.

#### Preparation of tumor cell colonies

Ht-29 cells (a human colorectal adenocarcinoma line) were obtained from the American Type Cell Culture Collection (Rockville, MD, USA) and cultured as previously described [7]. They were then seeded (*n* = 5 × 10^4^) onto a polystyrene disc (internal diameter 1 cm)—acting as a template for tumor cell colony growth—at the centre of Petri dish. When the cells reached semi-confluence, the template was removed and the tumor cell colony allowed to grow freely. Colonies were maintained at 37°C in a 5% CO_2_ atmosphere, changing half of the culture medium every three days.

#### Isolation of PMNs

PMN suspensions were obtained as previously described [17] from heparin-anticoagulated peripheral venous blood provided by volunteers. Cell viability, as measured by Trypan blue dye exclusion, always exceeded 95%. Total and differential cell counts were made using a Sysmex XE-2100 cell counter (Roche Spain, Barcelona, Spain).

#### Attracting PMNs to tumor cell colonies

Ht–29 cell colonies produced as above were set in growth medium in the wells of transwell devices. PMNs (*n* = 5 × 10^6^) were placed on a 3 *μ*m micropore mesh placed above each tumor colony; this pore size allows PMNs attracted to the tumor to pass through the mesh (simulating diapedesis) and approach the colony. All transwell devices thus prepared were then incubated overnight at 37°C in a 5% CO_2_ atmosphere. The cell colonies were then photographed over the next 100-200 h using an inverted microscope equipped with a contrast filter and a coupled digital camera. Photographs were scanned into a personal computer at a final resolution of 1.3 *μ*m/pixel, and the tumor borders hand traced to record changes in size.

## Results

### Free growth

**Fig 2:**
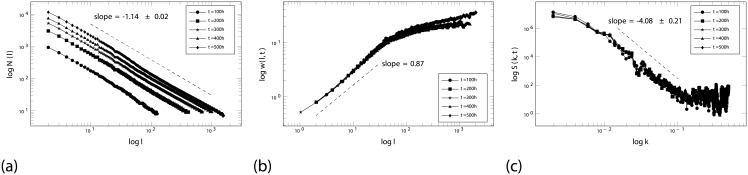
Characterization of free tumor growth as simulated by the new tool. (a) The value for the fractal dimension of the tumor border is 1.14. (b) The roughness of the tumor borders is quantitatively characterized by the value of the critical exponent of local roughness (*α*_*loc*_ = 0.87 ± 0.05). (c) The global roughness critical exponent for the tumor contour is provided by the latter’s power spectrum (value *α*_*glob*_ = 1.54 ± 0.10).

Fig 2 confirms the fractal nature of the tumor border and the information required to determine *α*_*glob*_ and *α*_*loc*_ (as a consequence of power law behavior, these critical exponents are obtained from the slopes of the presented graphs b and c). During the free growth simulation, the typical fractal dimension of the tumor border was seen to obey the expression *d*_*f*_ = 1.14 ± 0.02 (see Fig 2a). The fractal nature of the tumor border allows scaling techniques to be used to determine the growth dynamics of the tumoral mass. By analyzing the roughness of the border, a value of *α*_*loc*_ = 0.87 ± 0.05 for local roughness was obtained (see Fig 2b). From this it was determined that *d*_*f*_ + *α*_*loc*_ = 2.01, which coincides exactly with the value of self-afine interfaces in dimension 2 (the Euclidian dimension). *α*_*glob*_ was determined from the slope, m, of the plot of the power spectrum of the tumor border against the momenta (see Fig 2c). In this case, *m* = 2*α*_*glob*_ + 1 = 4.08 ± 0.21, providing a global roughness exponent of *α*_*glob*_ = 1.54 ± 0.10, which indicates the tumor border to be super-rough, i.e., to show anomalous scaling. Thus, the free growth simulation meets the criteria of the MBE universality class of dynamics, showing the tool to be UMTGD-compliant.

**Fig 3:**
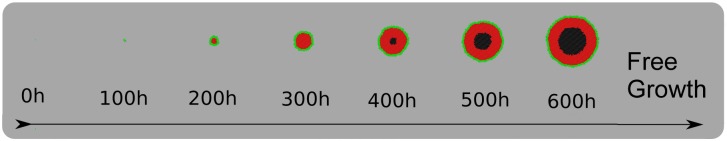
Image of the simulated tumor grown under free growth conditions, i.e., in the absence of any PMNs. This simulation corresponds to S1 Video.

**Fig 4:**
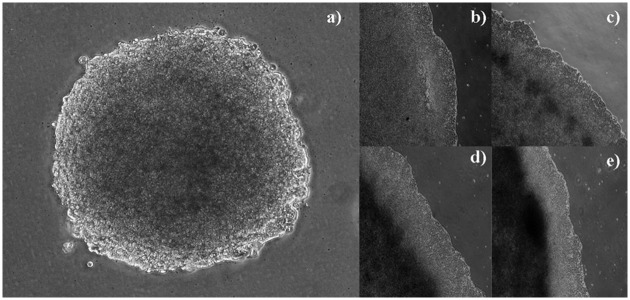
Image of an Ht-29 (human adenocarcinoma) tumor colony grown in the absence of PMNs. Note how the centre of the tumor (a) contains only necrotic cells (dark). Proliferating cells (bright) are observed only at the border, forming a thin rim. (a) Quiescent cells appear to be gray, forming a band between the necrotic core and the external rim of proliferating cells. (b) Magnified view of the tumor border before the time point shown in (a). (c) 60 h later, necrotic cells appear forming groups of cells. (d and e) 40 and 60 h later again, the necrotic core becomes denser and larger, as expected according to [7].

Fig 3 shows the tool’s representation of the spatial distribution of proliferative, quiescent and necrotic cells in a free growth scenario. Note that at the centre of the simulated tumor, where no space is available, the cells are in a necrotic state, as stipulated by the UMTGD [7]. Proliferating cells are only observed at the tumor border. Fig 4 confirms this to also be the case for the tumor colony grown in vitro in the absence of PMNs.

### Simulation of tumor-PMN interactions at different intensities of immune response

**Fig 5:**
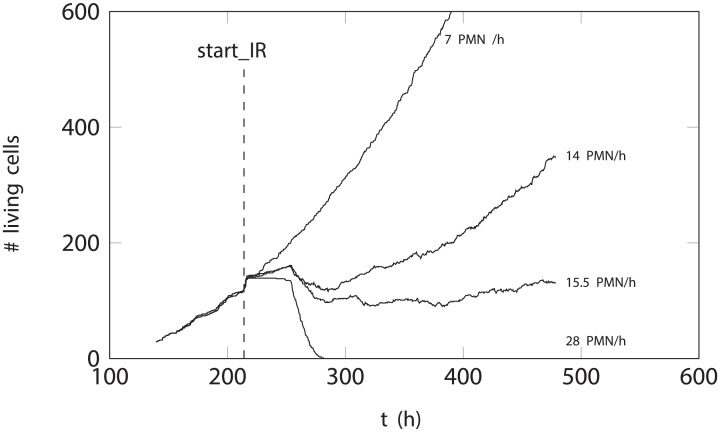
Change in size of simulated tumors when challenged by PMNs arriving at different rates. This image reveals the existence of an immunological threshold that separates immunodeficient from immunocompetent behavior, and thus tumor growth or regression. start-IR marks the point where PMN delivery began.

Fig 5 describes the effect of the different PMN arrival rates (immune response intensity) on tumor growth. As expected, the 7 PMN/h arrival rate allowed the tumor to continue to grow unchecked: the number of living cells increased over the entire simulation time.

**Fig 6:**
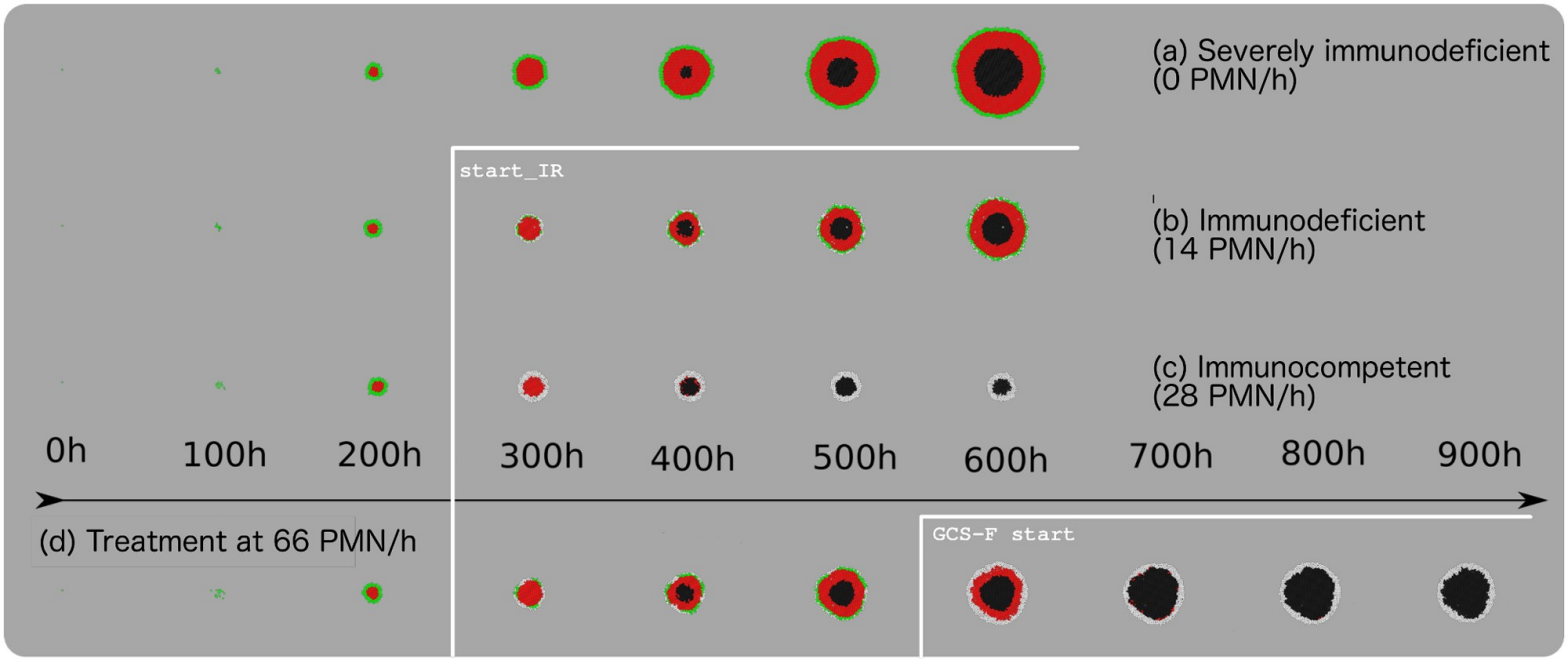
Simulation tool snapshots of tumor growth under different immune response conditions. For all simulations, proliferating cells are shown in green, quiescent cells in red, necrotic cells in black, and PMNs in white. (a) Tumor growth under a PMN arrival rate of 0 cells/h. Note how the tumor continues to grow. This represents what happens under conditions of chronic, extremely low-level peritumoral inflammation, i.e., a severely immunodeficient response. (b) Tumor growth under a PMN arrival rate of 14 cells/h. Note how the tumor continues to grow. This represents what happens under conditions of chronic, low-level peritumoral inflammation, i.e., an immunodeficient response. (c) Tumor growth under a PMN arrival rate of 28 cells/h. Note the change in the distribution of proliferative, quiescent and necrotic cells compared to that seen in the line above (14 PMN/h), and how the tumor regresses. These results represent what happens under conditions of acute peritumoral inflammation, i.e., an immunocompetent response. (d) Tumor growth under treatment conditions, i.e., a PMN arrival rate of 66 cells/h. Note how this induced acute peritumoral inflammation causes the regression of the tumor. *start*_*IR* marks the start of PMN delivery.

At 14 PMN/h, tumor growth was slower, but continued over the entire simulation period, i.e., the number of neutrophils arriving at the simulated tumor was still insufficient to prevent its growth. Fig 6 shows the distribution of proliferative, quiescent and necrotic cells to be similar to that seen for the 0 PMN/h scenario. S2 Video provides a visualization of this simulation. Fig 6 shows graphically the distribution of proliferative, quiescent and necrotic cells in the simulated tumor.

At 15.5 PMN/h, the tumor grew very little (see Fig 4). Fig 6 shows dividing cells to be largely absent from the tumor border, where the cells are now mostly quiescent.

At 28 PMN/h, the tumor did not grow. Nor did it grow at any higher rates of PMN arrival (data not shown). Fig 6 shows that, under these conditions of simulated acute peritumoral inflammation, the proliferative tumor cells have completely disappeared: the majority of cells appeared quiescent for some time, before they all became necrotic. S3 Video provides a visualization of this simulation.

### Treatment simulation

Fig 6 shows how the tumor, which grew until over 500 h, began to regress at the arrival of the PMNs at 66 cells/h. Maintaining this intensity of immune response led to the proliferating and quiescent cells all entering a necrotic state, with the proportion of dead cells becoming larger over time.

### PMN recruitment by tumor cell colonies

**Fig 7:**
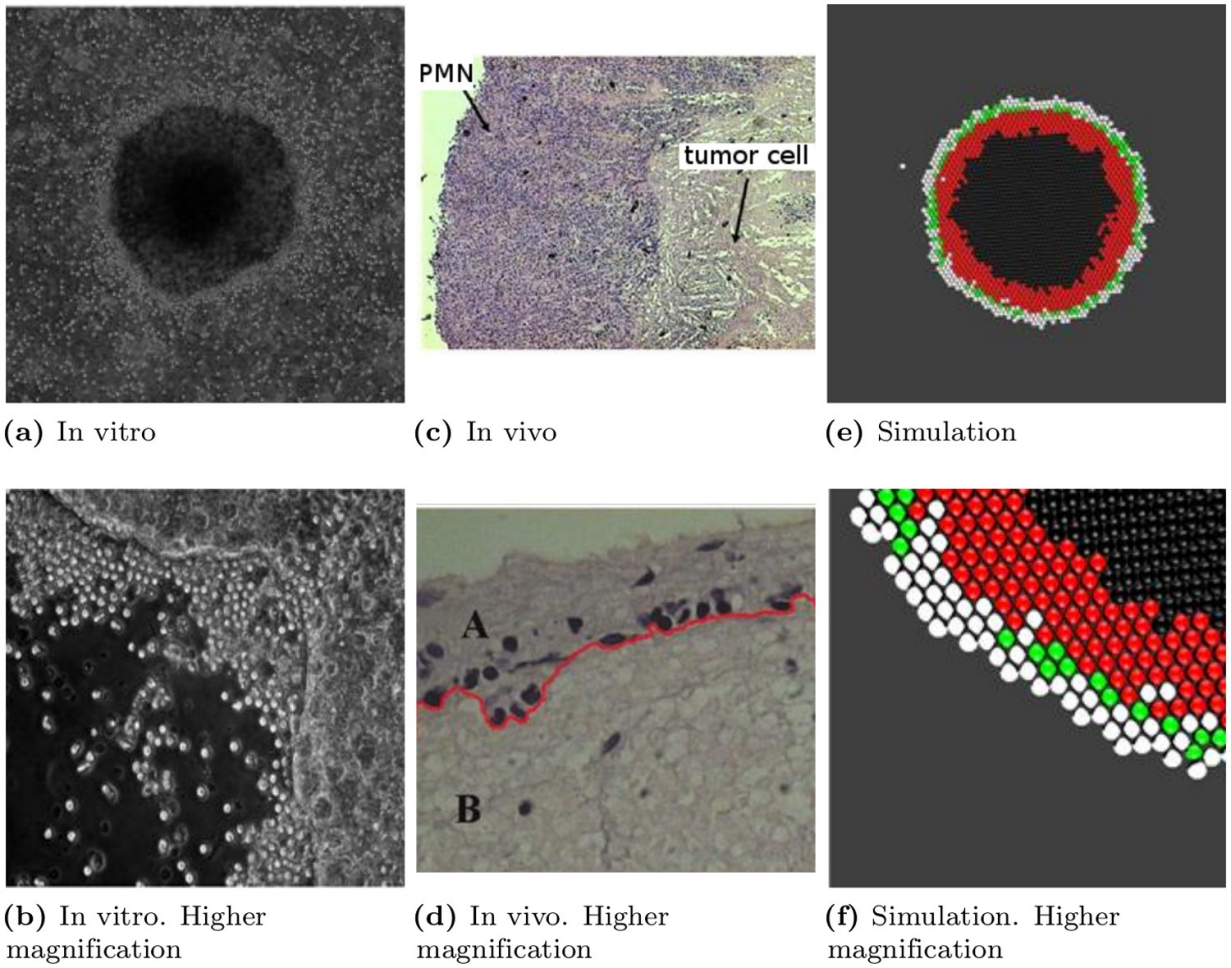
Acute peritumoral inflammation. (a) PMNs arriving at an Ht-29 tumor cell colony in a transwell plate during an immunocompetent response. (b) Higher magnification of above, showing PMNs occupying sites at the tumor cell colony border. (c) PMNs arriving at the border of an Ehrlich tumor in C57BL/6 mouse treated with 10 μg/kg/day G-CSF to stimulate the innate response. **(d)** High magnification of above, showing PMNs packaging the tumor. **(e)** Simulation of tumor under acute inflammation conditions. PMNs appear as white dots; other colors represent proliferative, quiescent and necrotic cells as above. **(f)** Higher magnification of above, showing PMNs packaging the tumor border, preventing tumor cell division. For the simulation, proliferating cells are shown in green, quiescent cells in red, necrotic cells in black, and PMNs in white. Note the similarities in the results for all three types of tumor.

Fig 7a and 7b show, at different magnifications, PMNs that have arrived at the tumor border during growth in the transwell plates. By way of comparison, Fig 7c and 7d show the same phenomenon captured in earlier in vivo work using an Ehrlich tumor implanted subcutaneously in a C57BL/6 mouse treated with 10 *μ*g/kg/day G-CSF to stimulate the innate response [18]. Fig 7e and 7f show the results obtained by the simulation tool during acute inflammation. The higher magnification pictures for all three examples show that the first PMNs to arrive occupy the free spaces at the tumor border, eventually forming a packaging layer that prevents any diffusion of new tumor cells at the tumor border. As long as the tumor remains thus packaged, no growth can occur. The proliferative cells become quiescent before eventually becoming necrotic.

## Discussion

### Validation of the simulation tool

The results of the free growth simulation show that the universal dynamics of tumor growth were obeyed, validating the proposed tool as an appropriate means of modeling tumor growth.

### The simulations performed reveal the existence of an immunological threshold

**Fig 8:**
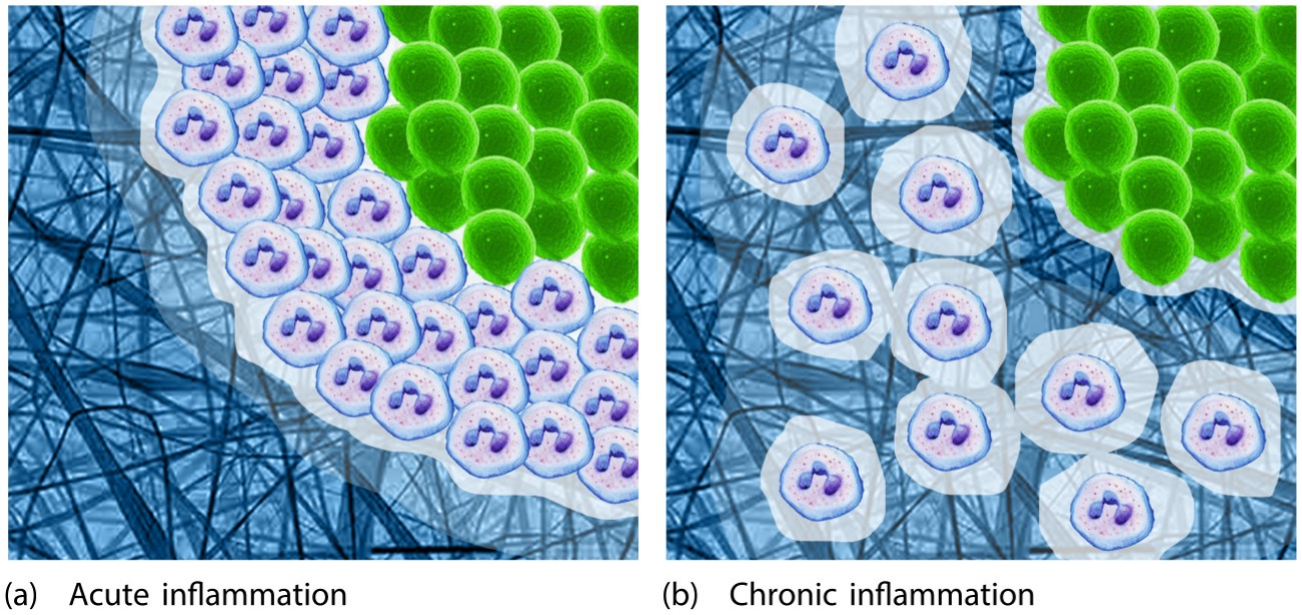
Difference between acute and chronic peritumoral inflammation. **(a)** Diagram reflecting acute peritumoral inflammation, i.e., the result of a response above the immunological threshold. Note how the PMNs (blue cells) package the tumor (green cells) tightly and occupy all the available space at the tumor border. There is no free space into which any new tumor cell can diffuse and settle. The tumor cells thus become quiescent and eventually necrotic. **(b)** Diagram reflecting chronic inflammation, i.e., the result of a response below the immunological threshold. The PMNs do not tightly package the tumor and space remains into which newly produce tumor cells can diffuse and settle. Indeed, the inherent cytolytic action of the PMNs could even increase the space available.

The simulations performed reveal the existence of a threshold arrival rate (immune response intensity) of approximately 14 PMN/h marking a boundary between a protumoral and antitumoral response (see Fig 5). At intensities below this “immunological threshold”, the simulated tumors continued to grow, while at intensities above it the distribution of proliferative, quiescent and necrotic cells shifted, indicating the tumor to have entered regression. In the treatment simulation too, the arrival of 66 PMN/h—well over the immunological threshold—led to the tumor becoming markedly necrotic and regressing. According to the dynamics that all tumors obey [7], the peritumoral inflammation induced by a response above the immunological threshold (acute inflammation) must cause the space at the tumor border—where new tumor cells need to diffuse and settle—to disappear (see Figs 7 and 8). Indeed, the PMNs around the tumor pack it tightly, filling this space, causing the tumor cells to become first quiescent and then enter a necrotic state, as confirmed in the present simulations and in earlier reports [3, 7, 18]. When the peritumoral inflammation induced is below the immunological threshold (chronic inflammation), the PMNs cannot make all the space at the tumor border unavailable; no packaging of the tumor occurs (see Fig 8). Indeed, the low numbers of PMNs that do arrive under such conditions could even increase the space available via their inherent cytolytic activity on the tissue surrounding the tumor, and their favoring angiogenesis [11]. Chronic inflammation can therefore promote tumor growth and even favor the formation of metastases [11, 12].

### Can increasing the number of circulating PMNs induce a strong antitumoral effect in vivo? Observations in animal models

The present simulations suggest that inducing strong and sustained peritumoral inflammation via the use of, for example, G-CSF, could be used as a treatment to invert tumor growth. G-CSF is a cytokine essential to the production and differentiation of PMNs; its antitumoral effects have been recorded in a number of studies. Colombo et al. [19] reported an increased number of PMNs around implanted adenocarcinomas in transgenic mice carrying the human G-CSF gene. A similar effect was reported by Cavallo et al. [20] in the same mouse model in which the animals carried the human gene for IL-2, which also increases PMN production. Kokura et al. [21] showed PMNs to increase in number after the arterial administration rhG-CSF in a whole body hypethermia rat model of AH109A carcinoma. Tamamori et al. [22] reported an antitumoral effect of a monoclonal antibody against pancreatic cancer in BALB/c nude mice, but this appeared much increased when these animals simultaneously received human G-CSF. Similarly, van Spriel et al. [23] reported an antitumoral effect of a monoclonal antibody against melanoma, but this also appeared increased when G-CSF was administered and the number of circulating PMNs consequently increased. More recently, Siders et al. [24] reported that the administration of G-CSF plus alemtuzumab achieved 100% survival in mice with lymphoma, compared to 60% in those administered only the antibody. In fact, the administration of human recombinant G-CSF on its own has been reported to have a strong antitumoral effect. Matsumoto et al. [25] reported the inhibition of growth in liver and lung metastases in murine models of melanoma, lung cancer and lymphoma after G-CSF administration increased the number of circulating PMNs by just three fold.

Similar results were obtained [18] in a mouse model of fibroehrlich cell line by daily administration of granulocyte macrophage colony stimulating factor (GM-CSF) for eight weeks, in which the total elimination of tumors was achieved in 20% of mice, as well as a general increase in survival time. The death ratio was 15.76 times higher than in the treated group (*p* = 0.004) and a high tumor necrosis (around 80% − 90%) of the rest of tumors was achieved.

The fact that the considerable increase of tumor necrosis occurs in the innermost part of the tumor reinforces the argument that the lack of space for duplication increases the transition from a proliferative state of cells to a quiescent one and from a quiescent to a necrotic state. If the main action was cytotoxic, necrosis would appear in the outermost area of the tumor. This fact of cell death can also be seen in the *in vitro* culture of tumor cell colonies (even those that grow in monolayer [7]) in which cell death occurs in the innermost regions of the colonies, despite having all the cells in the colonies the same access to nutrients.

### G-CSF and GM-CSF as antitumoral agents in the clinical setting

Following the establishment of the universal tumor growth theory, the complete regression of a liver tumor was achieved in a patient who was compassionately administered G-CSF [26]. The administration of GM-CSF was also reported to have an antitumoral effect in phase II trials involving patients with advanced melanoma [27], breast cancer and female genital tract carcinoma [28]. G-CSF was suspected of having a similar effect in a randomized, double-blind clinical trial to determine whether it reduced the mucositis associated with several weeks of post-operative radiotherapy for head and neck cancer [29]. Although it proved ineffective in this regard, and the trial was ended early, it was later noticed that the 5-year survival rate of those who had received G-CSF was 84% compared to 47% for those who did not. Certainly, the former patients had a much higher number of PMNs when this was measured on day 50 (24.100 ± 2700/mm^3^ compared to 4.100 ± 1.500/mm^3^ in those who received no G-CSF). It is interesting to note here that in a study involving patients with different tumors, in which PMNs were assumed to be protumoral in their effects, and were therefore removed from the blood, no clinical improvements were seen [30]. Further clinical trials should be performed to confirm whether the induction of strong and sustained peritumoral inflammation causes tumors to regress. Since tumor growth dynamics are universal, these trials could involve multiple tumor types. The present simulations suggest, however, that any change between a response above and below the immunological threshold—as might occur in patients with cancer due to stress etc. [31]—could lead to periods of tumor growth and regression, highlighting the need not to suspend G-CSF treatment too early. The risk of possible side effects when maintaining a level of peritumoral inflammation above the proposed immunological threshold would appear acceptable in the treatment of cancer.

Treatment with G-CSF is associated with very few adverse events [29]. Intense circulating neutrophilia would be safe, based on the clinical reports previously cited and some older reports on constitutive neutrophilia [32, 33] and certainly the pharmaceutical companies that market G-CSF report no problems to arise at 100 × 10^9^ PMNs/L, observed in at least 2% of the patients treated for neutropenia ([34]). Other reports in which G-CSF has been used to raise PMNs levels 56,000 ± 16,000/mm^3^ suggest this not to be associated with adverse circulatory or cardiovascular effects [35].

### Acute inflammation and anti-tumoral effects

The classical works of William Coley, pioneer in cancer immunotherapy, and some recently discovered (e.g. [36]) proved the anti-tumoral effects of the administration of pyrogenic bacterial products without infectious capabilities. The antitumoral effect was linked to the regular administration of a toxin (composed of extracts of Gram-positive bacteria Streptococcus pyogenes and dead Gram-negative Serratia marcescens) for prolonged periods, causing high fever and shivers and, in all likelihood, intense neutrophilia [37].

Several independent observations of the link between acute infection (inevitably accompanied by neutrophilia) and higher survival rates to cancer are also present in the literature. This is the case of [38], who published a 5 years survival rate of 50% of lung cancer removal surgery patients with post-operative empiema complications, compared to the 18% survival of the control group (with no empiema or infection).

Bladder cancer is another very interesting clinical context, in which local instillation of Bacillus Calmette-Guérin (BCG) induces a strong immune response and has been the standard of care for patients since 1977 (see [39, 40]). This is one of the most successful immunotherapy for solid tumors over the last decades. A strong negative correlation has been observed between leukocyturia levels (massively dominated by PMNs) and tumoral recurrence. Patients without tumoral recurrence show twice as much leukocytes in urine than those with tumor progression [41, 42].

## Conclusions

This work reports a simple informatic tool based on well founded biological principles that reproduces key aspects of tumor growth, and explains how this growth changes under different intensities of the innate immune response. The simulation results reveal the existence of an immunological threshold that explains previous clinical reports in which peritumoral inflammation sometimes appeared to be protumoral and sometimes antitumoral in its effects. Simulated tumors challenged with an intense PMN arrival rate regressed in size, and real tumors have been reported by different authors to behave similarly under such conditions. These findings suggest that, based on the universal growth dynamics of solid tumors, maintaining strong peritumoral inflammation with PMNs via treatment with compounds such as G-CSF, could provide a universal treatment.

## Supporting information

S1 Video Growth of a simulated tumor without presence of PMNs. Green, red and black represent proliferative, inhibited and necrotic cells respectively.
(MP4)

S2 Video Growth of a simulated tumor under a PMN (white) arrival rate of 14 PMN/h. Green, red and black represent proliferative, inhibited and necrotic cells respectively.
(MP4)

S3 Video Growth of a simulated tumor under a PMN (white) arrival rate of 28 PMN/h. Green, red and black represent proliferative, inhibited and necrotic cells respectively. Note how the distribution of cells shifts towards there being more quiescent and necrotic cells and fewer proliferative cells.
(MP4)

S1 Data Raw data from the simulation. File containing the position and radius of particles at simulated times. A detailed description file is provided alongside the data.
(ZIP)

S2 Data Data from graphs. Data points for the represented graphs are provided. A detailed description file is provided alongside the data.
(ZIP)
